# Transfer of *Candidozyma auris* to Healthcare Personnel Gloves and/or Gowns From Patients and the Environment: A Multicenter Cohort Study of 109 Patients

**DOI:** 10.1093/ofid/ofag582

**Published:** 2026-09-08

**Authors:** Lyndsay M O’Hara, Sarah E Sansom, Mary K Hayden, David P Calfee, Susan S Huang, Mary Bahr-Robertson, Lisa Pineles, J Kristie Johnson, Michelle Newman, Raveena D Singh, Raheeb Saavedra, Unwana Umana, Anthony D Harris

**Affiliations:** Department of Epidemiology & Public Health, University of Maryland School of Medicine, Baltimore, Maryland, USA; Division of Infectious Diseases, Rush University Medical Center, Chicago, Illinois, USA; Division of Infectious Diseases, Rush University Medical Center, Chicago, Illinois, USA; Division of Infectious Diseases, Weill Cornell Medicine, New York, New York, USA; Division of Infectious Diseases, University of California, Irvine School of Medicine, Irvine, California, USA; Department of Epidemiology & Public Health, University of Maryland School of Medicine, Baltimore, Maryland, USA; Department of Epidemiology & Public Health, University of Maryland School of Medicine, Baltimore, Maryland, USA; Institute for Health Computing, University of Maryland, North Bethesda, Maryland, USA; Department of Epidemiology & Public Health, University of Maryland School of Medicine, Baltimore, Maryland, USA; Department of Epidemiology & Public Health, University of Maryland School of Medicine, Baltimore, Maryland, USA; Division of Infectious Diseases, University of California, Irvine School of Medicine, Irvine, California, USA; Division of Infectious Diseases, University of California, Irvine School of Medicine, Irvine, California, USA; Division of Infectious Diseases, Weill Cornell Medicine, New York, New York, USA; Department of Epidemiology & Public Health, University of Maryland School of Medicine, Baltimore, Maryland, USA; Institute for Health Computing, University of Maryland, North Bethesda, Maryland, USA

**Keywords:** acute care, *Candida auris*, *Candidozyma auris*, emerging pathogens, infection prevention

## Abstract

**Background:**

*Candidozyma auris* is an emerging pathogen. We sought to understand risk factors for *C auris* transmission in acute care hospitals, using transfer of *C auris* to healthcare personnel (HCP) gloves and/or gowns as a surrogate measure of transmission potential.

**Methods:**

This was a prospective, multicenter cohort study of HCP gloves and gowns cultured for *C auris* during routine patient care. Patient samples from 5 body sites and environmental surfaces were also cultured for *C auris*.

**Results:**

Among 109 *C auris*–colonized patients, we found *C auris* transfer to HCP gloves and/or gowns in 29% of 1083 HCP interactions. Transfer was highest among nursing assistants/patient care technicians (odds ratio [OR], 5.2 [95% confidence interval {CI}, 2.4–11.6]), occupational/physical therapists (OR, 4.8 [95% CI, 1.4–16.7]), and respiratory therapists (OR, 4.7 [95% CI, 1.9–11.7]). Transfer was high when HCP touched the patient (OR, 3.32 [95% CI, 2.3–4.9]) compared to touching only an environmental surface. Patients who were colonized at 5 body sites were 15 times more likely to transfer *C auris* to HCP gloves and/or gown than those colonized at only 1 site (OR, 15.1 [95% CI, 7.5–30.5]).

**Conclusions:**

This study demonstrates that transfer of *C. auris* to HCP gloves and/or gowns, a surrogate for transmission potential, occurs frequently during patient care. Certain HCP and patient care tasks or interactions are associated with higher rates of transfer. Strategies to interrupt transfer of *C auris* to HCP during patient care are needed to prevent *C auris* transmission in healthcare facilities.

## Teaser

This multicenter study found that transfer of *Candidozyma auris* to healthcare personnel gloves and/or gowns, a surrogate for transmission potential, occurred in 29% of 1083 interactions. Certain healthcare personnel and patient care tasks are associated with higher rates of transfer.

*Candidozyma auris* (formerly *Candida auris*) has been identified as an urgent threat by the Centers for Disease Control and Prevention [1]. The number of cases of *C auris* have steadily increased in the United States and worldwide [1, 2]. The first case of *C auris* in the United States was reported in 2016; by 2023, there were 4514 clinical cases reported [3]. *Candidozyma auris* is associated with significant morbidity and mortality and spreads rapidly in healthcare facilities [3, 4]. Our previous work in the acute care setting with patients colonized or infected with methicillin-resistant *Staphylococcus aureus* (MRSA) and carbapenem-resistant Enterobacterales (CRE) suggests that certain healthcare personnel (HCP) roles, patient care interactions, and increased patient bacterial bioburden are associated with higher rates of transfer of these organisms to HCP gloves and gowns [5–7]. The overall aim of this study was to understand risk factors for *C auris* transmission in the acute care setting, using transfer of *C auris* to HCP glove and/or gown as a surrogate measure of transmission potential. This study aimed to evaluate the following factors associated with the transfer of *C auris* to HCP gloves and/or gowns: (1) HCP role; (2) touching the patient or the environment while performing patient care tasks or during other HCP–patient interactions; (3) patient bioburden of *C auris*; (4) concentration of chlorhexidine gluconate (CHG) on the patient's skin; and (5) proportion of room environmental surfaces contaminated with *C auris.*

## MATERIALS AND METHODS

### Study Design and Participants

We performed a prospective, multicenter observational cohort study between 8 July 2022 and 1 May 2024. Patients with a current or prior history of *C auris* were enrolled at 9 acute care hospitals in Baltimore, Maryland; Irvine, California; Chicago, Illinois; and New York, New York. The study was approved as a quality improvement project in Irvine, California. All other facilities received institutional review board (IRB) approval (IRB protocol numbers: Weill Cornell Medical College: 1610017615; Rush University: 21120603-IRB01; University of Maryland, Baltimore: HP-00066759).

### Data Collection

Upon enrollment, Copan ESwabs (Copan, Murrieta, CA, USA) were used to obtain samples from patients’ bilateral axillae, groin/inguinal creases, finger webs, anterior nares, and stool. When stool collection was not possible, a perirectal sample was obtained. All swabs and stool samples were sent to a single laboratory at the University of Maryland for processing. In clinical areas where CHG bathing was performed daily, additional swab samples were obtained from the axillae and inguinal areas using premoistened cotton swabs (Bio-Swab Pre-Filled Cotton Tipped Applicator, Arrowhead Forensics, Lenexa, KS, USA) to estimate residual post-bath CHG concentrations on the skin. Samples were also obtained from 8 environmental surfaces in the patient's room (bedside table, bedrail, vital sign monitor, sink, IV pump, supply cart, floor, and call button) using sponge sticks (3M Sponge-Stick with 10 mL neutralizing buffer) and a 10 × 10-cm sterile stencil.

The research team observed 10 routine patient–HCP interactions in each patient room, recording care tasks and all items touched by the HCP using a standardized data collection form. Prior to room exit but before removal, HCP gloves and gown were sampled separately using BD BBL CultureSwab (Becton Dickinson, Sparks, MD, USA). Using a twirling motion, the swab was rubbed along each finger and the palm of both gloved hands. HCP gowns were sampled twice along both forearms and then in a “W” pattern along the beltline as done in previous studies [5, 6]. A unique set of glove and gown swabs was obtained for each interaction; however, HCP did not have to be unique, meaning that if a HCP entered the room on 2 different occasions during the observation period, they could be swabbed twice. The primary outcome was a surrogate measure for transmission potential defined as transfer of *C auris* to HCP glove and/or gown [5–7]. See the Supplementary Materials for standard operating procedures and observation forms.

### Laboratory Methods

#### Patient Samples

Patient samples in ESwab Amies transport media were directly inoculated onto HardyCHROM Candida + auris agar (Hardy Diagnostics, Santa Maria, CA, USA) and serially diluted (1:10) in Butterfield buffer for quantitative culture in triplicate with a plating volume of 100 µL. Following incubation at 35°C for 48 hours, cultures with 30–300 presumptive *C auris* colonies were selected for enumeration. Colony counts were averaged across replicates and multiplied by the dilution factor to calculate colony-forming units per milliliter (CFU/mL). In addition, an aliquot of patient sample was inoculated into Salt Sabouraud Dulcitol Broth with chloramphenicol and gentamicin (Hardy Diagnostics) and incubated at 40°C for 5 days to optimize culture sensitivity. Positive enrichment broths were plated to HardyCHROM Candida + auris.

For CHG levels, a colorimetric, semiquantitative method was used to estimate CHG concentrations as previously described [8–10].

#### Environmental Samples

Sponge sticks were placed in Stomacher bags with 40 mL of phosphate-buffered saline with Tween 20 and homogenized for 1 minute at 260 rpm using a Stomacher (Seward Inc, Bohemia, NY, USA). Sample homogenates were concentrated by centrifuging at 3500*g* for 15 minutes, and 100 μL was inoculated into Salt Sabouraud Dulcitol Broth with chloramphenicol and gentamicin and incubated at 40°C for 5 days. Positive enrichment broths were plated and assessed for *C auris* as described above.

#### HCP Glove and Gown Samples

Swabs were inoculated directly into Salt Sabouraud Dulcitol Broth with chloramphenicol and gentamicin and incubated at 40°C for 5 days. Positive enrichment broths were processed as described above.

### Statistical Analyses

Frequencies and proportions were calculated to describe the transfer of *C auris* onto HCP gloves and/or gown for each factor of interest. Patient *C auris* burden (x + 1) was log transformed and expressed in log_10_ CFU/mL or colony-forming units per centimeter squared (CFU/cm^2^). Median and interquartile range (IQR) were calculated to describe patient *C auris* burden at 6 body sites and the concentration of CHG on the skin expressed as micrograms per milliliter (μg/mL). We estimated the following associations with HCP glove and/or gown contamination: HCP role, contact with patient and/or environmental domain, and patient *C auris* bioburden. We also estimated the associations between CHG concentrations on the axillae and inguinal areas with patient *C auris* burden at these same sites. For assessment of *C auris* transfer as a marker for potential transmission, all models were built separately using logistic regression fit by generalized estimating equations with an exchangeable correlation matrix to account for within-patient correlation. Simple linear regression was used to test if CHG levels on the skin predicted *C auris* bioburden at the same skin site. All analyses were conducted using SAS version 9.4 software (SAS Institute, Cary, NC, USA).

## RESULTS

### Cohort Characteristics

A total of 137 patients with a history of *C auris* were enrolled in the study. Twenty-eight of these patients were negative for *C auris* at all body sites upon enrollment and were excluded from analyses. There were 1083 observed patient-HCP interactions for the 109 remaining *C auris*–colonized patients. Clinical characteristics of patients were as follows: 78 (72%) had a wound, 54 (50%) had an artificial airway, 44 (40%) had a central venous catheter, 39 (36%) had an indwelling urinary catheter, 24 (22%) had diarrhea documented at the time of enrollment, 12 (11%) had a surgical drain, 11 (10%) had a rectal tube, 10 (9%) had a nasogastric tube, and 5 (5%) had a chest tube. Seven patients (6%) had none of these characteristics at the time of enrollment.

### Transfer of *C auris* to HCP Gloves and/or Gown

Overall, transfer of *C auris* to HCP gloves occurred in 271 (25%), gowns in 132 (12%), and gown and/or gloves in 316 (29%) of 1083 observations. When stratified by study site, the rate of *C auris* transfer to HCP glove and/or gown ranged from 18% to 32% (Table 1). Among the 33 patients who were in the intensive care unit (ICU) at the time of enrollment, transfer occurred in 98 of 328 observations (30%). Among the 76 patients who were in non-ICU areas at the time of enrollment, transfer of *C auris* to HCP gloves and/or gowns occurred in 218 of 755 observations (29%) (Table 1).

**Table 1.** Description of Study Sites, Patient Care Location, Patient Characteristics, and Associated Rates of *Candidozyma auris* Transfer to Healthcare Personnel Gowns and/or Gloves During 1083 Patient–Healthcare Personnel Interactions

Characteristic	Unique Patients (N = 109), No. (%)	Transfer of *C auris* to Glove and/or Gown (N = 1083 Swabs), no./No. (%)
Study site		
Site 1	39 (35.8)	121/390 (31.0)
Site 2	12 (11.0)	22/119 (18.5)
Site 3	32 (29.4)	91/318 (28.6)
Site 4	26 (23.9)	82/256 (32.0)
Patient care location		
Intensive care unit	33 (30.3)	98/328 (29.9)
Non–intensive care unit	76 (69.7)	218/755 (28.9)
Patient characteristics		
Wound	78 (71.6)	244/774 (31.5)
Artificial airway	54 (49.5)	195/539 (36.2)
Central venous catheter	44 (40.4)	124/438 (28.3)
Indwelling urinary catheter	39 (35.8)	117/387 (30.2)
Diarrhea	24 (22.0)	81/240 (33.8)
Surgical drain	12 (11.0)	40/117 (34.2)
Rectal tube	11 (10.1)	42/110 (38.2)
Nasogastric tube	10 (9.2)	40/99 (40.4)
Chest tube	5 (4.6)	19/50 (38.0)

### HCP Role

Transfer of *C auris* to gloves and/or gowns varied by HCP role. Nursing assistants/patient care technicians had the highest odds of *C auris* transfer, with 37% of all interactions resulting in transfer (odds ratio [OR], 5.2 [95% confidence interval {CI}, 2.4–11.6]) when compared to environmental services personnel among whom *C auris* was transferred in 10% of observations. The second highest odds of transfer were observed among respiratory therapists and physical/occupational therapists, both resulting in transfer of *C auris* 35% of the time (OR, 4.8 [95% CI, 1.4–16.7] and OR, 4.7 [95% CI, 1.9–11.7], respectively) (Table 2).

**Table 2.** Healthcare Personnel Role and Adjusted Odds Ratios for Transfer of *Candidozyma auris* to Gloves and/or Gown

Healthcare Personnel Role	Transfer of *C auris* to Glove and/or Gown, no./No. (%)	aOR (95% CI)
Nursing assistant/patient care technician	59/159 (37.1)	5.2 (2.4–11.6)
Occupational/physical therapist	6/17 (35.3)	4.8 (1.4–16.7)
Respiratory therapist	22/63 (34.9)	4.7 (1.9–11.7)
Nurse	144/455 (31.6)	4.1 (1.9–8.8)
MD/NP/PA	53/214 (24.8)	2.9 (1.3–6.5)
Other^a^	24/95 (25.3)	2.9 (1.3–7.1)
Environmental services technician	8/79 (10.1)	Ref

Abbreviations: aOR, adjusted odds ratio (adjusted for clustering by patient); CI, confidence interval; MD, medical doctor; NP, nurse practitioner; PA, physician assistant.

^a^Other includes patient transport, pastoral care, electrocardiography/cardiac technician, phlebotomist, radiology/X-ray technician, speech language pathologist, mobility technician, dietary/nutritionist, pathology technician, social worker.

### HCP Interactions With the Patient and the Environment

**Figure 1.**
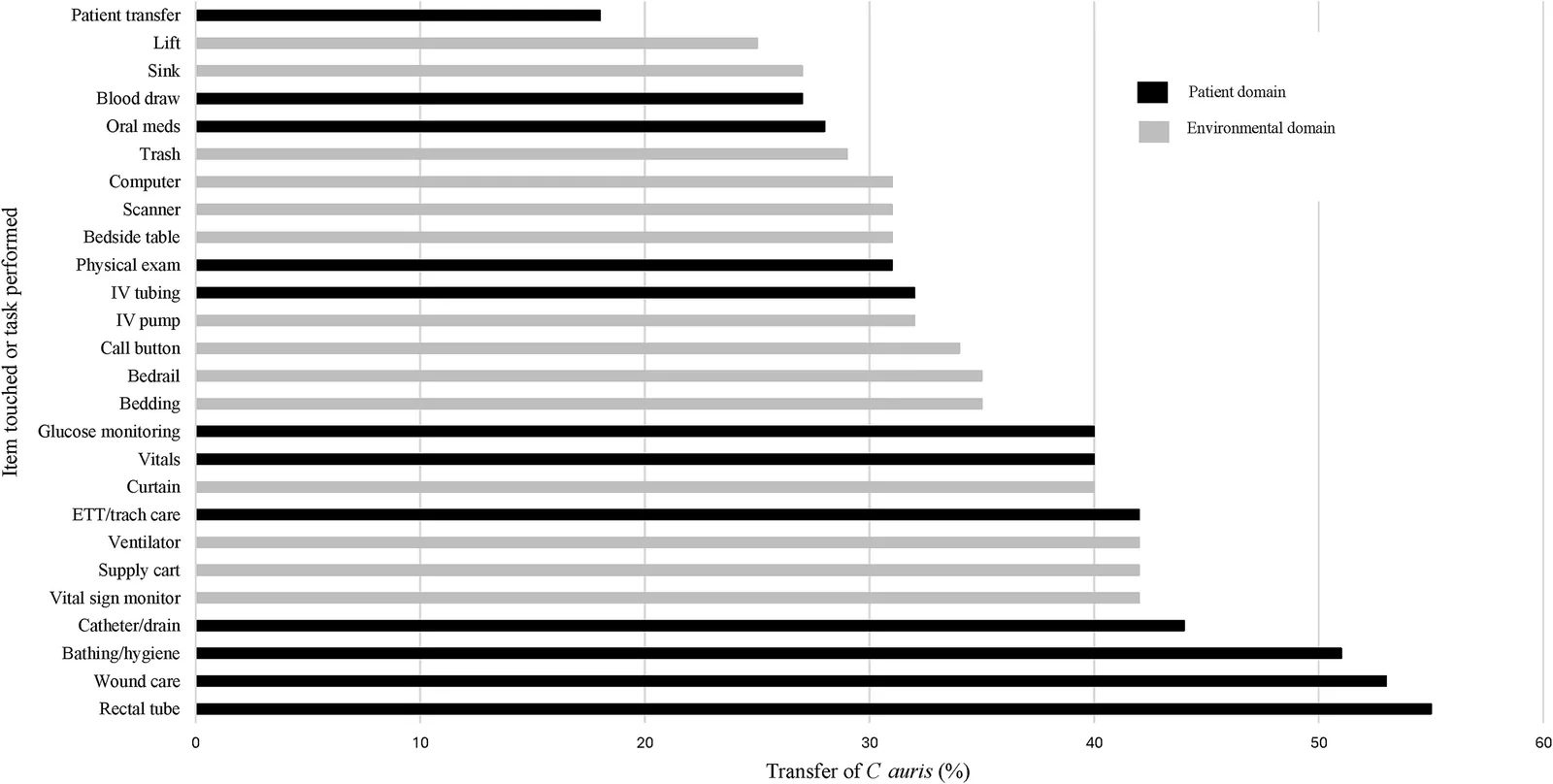
Transfer of *Candidozyma auris* to healthcare personnel gloves and/or gown by individual items touched or task performed. Abbreviations: ETT, endotracheal tube; IV, intravenous.

Environmental surfaces with the highest rate of *C auris* transfer to HCP gloves and/or gowns upon HCP contact were the vital sign monitor, supply cart, and ventilator (42% each). Patient care activities with the highest rate of transfer included handling the rectal tube (55%) and providing wound care (53%) (Figure 1). Any interactions that involved touching the patient (either the patient only or the patient and the environment) resulted in *C auris* transfer to HCP glove and/or gown in 282 of 829 (34%) observations (OR, 3.32 [95% CI, 2.25–4.90]). If the HCP touched only the environment, *C auris* was transferred to the gloves and/or gown in 34 of 253 (13%) observations (OR, 0.23 [95% CI, .05–.97]) (Table 3).

**Table 3.** Association Between Touching Patient and Environmental Domains and Transfer of *Candidozyma auris* to Healthcare Personnel Gloves and/or Gown

Domain Touched	Transfer of *C auris* to Glove and/or Gown, no./No. (%)	aOR (95% CI)
Patient only *OR* patient + environment	282/829 (34.0)	3.32 (2.25–4.90)
Environment only	34/253 (13.4)	0.23 (.05–.97)

Abbreviations: aOR, adjusted odds ratio (adjusted for clustering by patient); CI, confidence interval.

### Environmental *C auris* Burden

**Figure 2.**
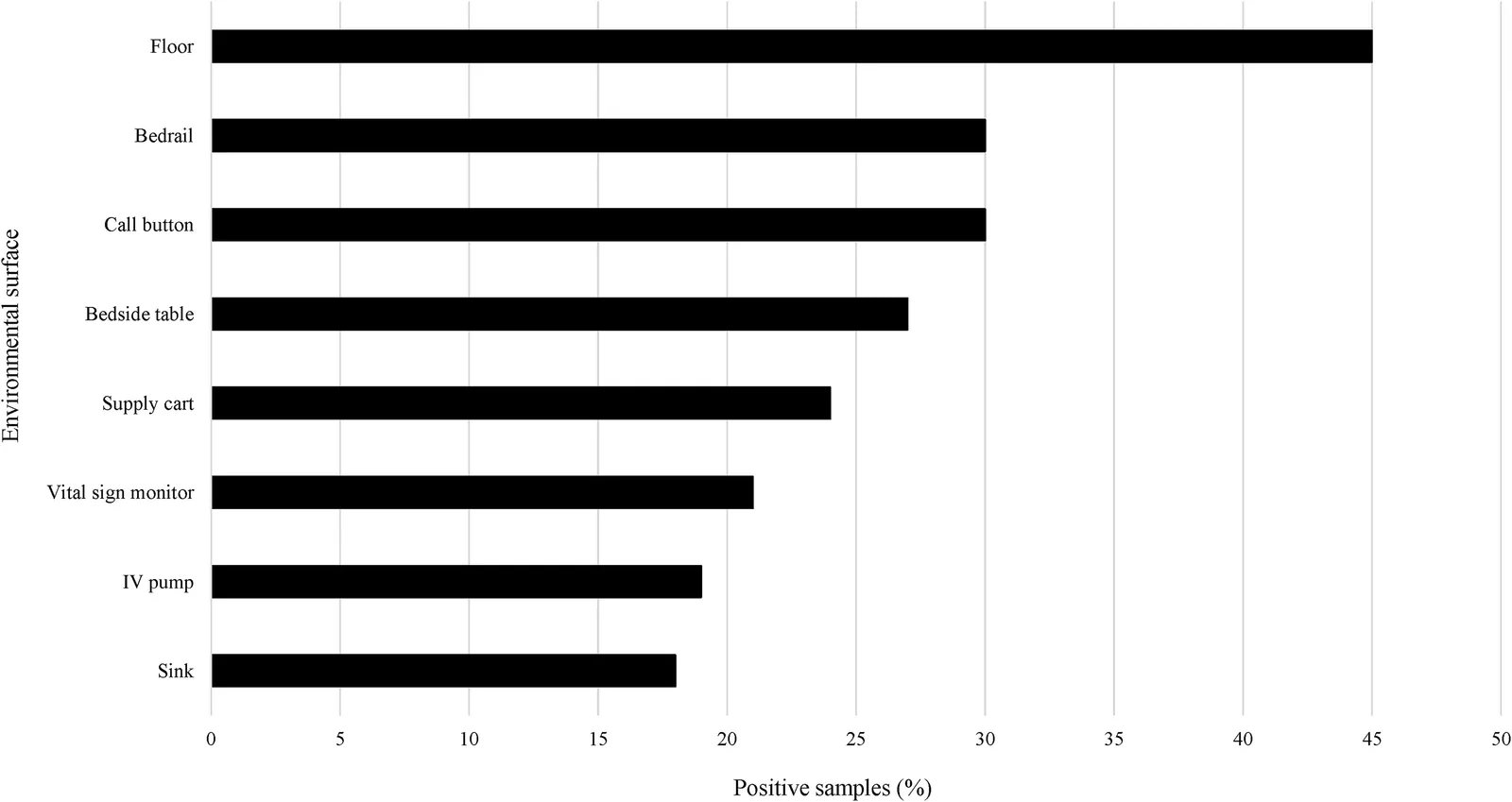
Percentage of environmental surfaces that were culture positive for *Candidozyma auris*. Abbreviation: IV, intravenous.

Environmental surfaces were frequently contaminated, with *C auris* recovered most frequently from the floor (45% of rooms sampled), followed by the call button and the bedrail (both 30%) (Figure 2).

### Patient *C auris* Burden

The most frequently colonized body sites were the fingers (82/107 [77%]) and the perirectal area (55/71 [77%]). *Candidozyma auris* was detected in 24 of 32 (75%) stool samples obtained, in 78 of 107 (73%) groin samples, 72 of 109 (66%) axilla samples, and 67 of 109 (61%) nares samples. Most patients were colonized with *C auris* at multiple body sites: 36 (33%) of the patients were colonized at 5 body sites, 28 (26%) at 4 body sites, 14 (13%) at 3 body sites, 14 (13%) at 2 body sites, and 17 (16%) at only 1 body site that was tested. The number of body sites colonized with *C auris* was associated with transfer of *C auris* to HCP gloves and/or gown. Patients who were colonized at 5 body sites were 15 times more likely to transfer *C auris* to HCP gloves and/or gown than those who were colonized at only 1 body site (OR, 15.1 [95% CI, 7.5–30.5]) (Table 4). The proportions of positive samples for each combination of patient body sites cultured are shown in Supplementary Table 1.

**Table 4.** Association Between Number of Positive Body Sites and Transfer of *Candidozyma auris* to Healthcare Personnel Gloves and/or Gown

No. of Positive Patient Samples^a^	Patients, No. (%) (N = 109)	Transfer of *C auris* to Glove and/or Gown, no./No. (%) (N = 1083)	aOR (95% CI)
1	17 (15.5)	9/168 (5.4)	Ref
2	14 (12.8)	12/139 (8.6)	1.7 (.7–4.1)
3	14 (12.8)	22/137 (16.1)	3.4 (1.5–7.6)
4	28 (25.7)	107/279 (38.4)	11.0 (5.4–22.4)
5	36 (33.0)	166/360 (46.1)	15.1 (7.5–30.5)

Abbreviations: aOR, adjusted odds ratio (adjusted for clustering by patient); CI, confidence interval.

^a^Any combination of all sites sampled, which included axilla, groin, finger webs, nares, perirectal, and stool.

**Figure 3.**
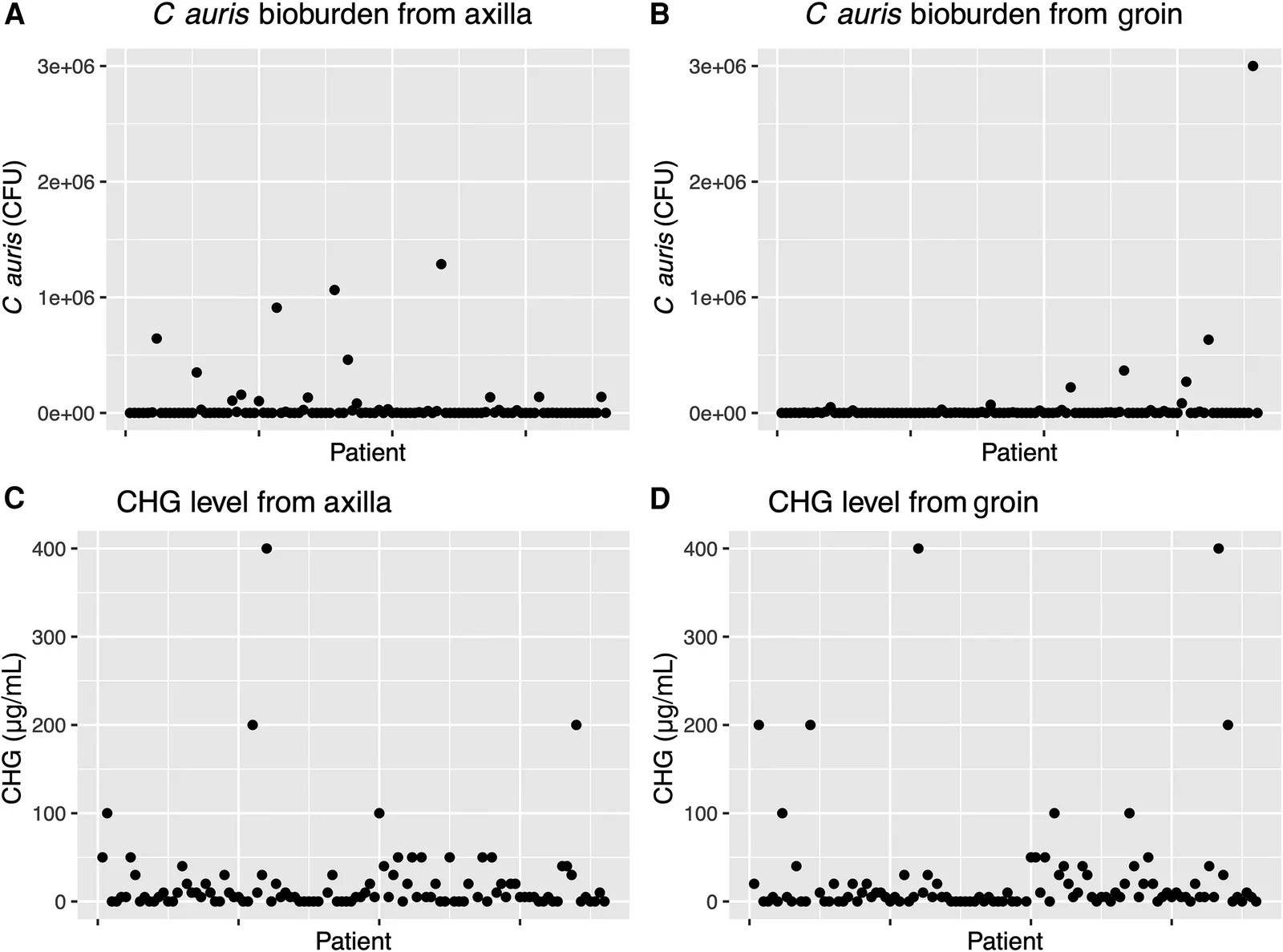
*Candidozyma auris* bioburden (colony-forming units [CFU/mL]) recovered from the axilla (*A*) and groin (*B*) and chlorhexidine gluconate (CHG) levels (μg/mL) recovered from the axilla (*C*) and groin (*D*).

The highest *C auris* recovery was from the patient finger webs (median, 238 [IQR, 0–18 675] CFU/mL), followed by the perirectal area (median, 93 [IQR, 0–1409] CFU/mL), inguinal creases (median, 55 [IQR, 0–1645] CFU/mL), axillae (median, 41.5 [IQR, 0–4992] CFU/mL), stool (median, 28 [IQR, 0–1230] CFU/mL), and nares (median, 11.5 [IQR, 0–3404] CFU/mL). Figure 3 illustrates the *C auris* bioburden (CFU/mL) recovered from the axilla (Figure 3A) and groin (Figure 3B) and CHG levels (μg/mL) recovered from the axilla (Figure 3C) and groin (Figure 3D). Higher CHG concentration on the groin was associated with lower *C auris* bioburden on the groin (β = −.015, *P* = .01), but CHG concentration on the axilla was not associated with *C auris* bioburden on the axilla (β = −.003, *P* = .73). Total *C auris* bioburden on the axilla (β = .03, *P* =< .0001) and finger (β = .01, *P* = .01) was associated with *C auris* transfer to HCP glove and/or gown, but that of the groin, nares, perirectal, and stool burden was not.

## DISCUSSION

This multicenter cohort study demonstrates that transfer of *C auris* to HCP gloves and/or gowns, a surrogate for transmission potential, occurs frequently during routine patient care. Certain HCP roles and specific patient care tasks or interactions are associated with higher rates of transfer. In addition, the number of patient body sites with detectable *C auris* and patient *C auris* bioburden were associated with transfer to gloves and/or gowns.

The methods we used in this study were nearly identical to previous multicenter studies on MRSA and CRE [5, 6]. However, the rate of *C auris* transfer to HCP gloves and/or gowns was higher, at 29%, than that observed for MRSA and CRE, which were estimated at 16% and 10% respectively [5, 6]. Reasons for these differences in transfer rates of organism are not known. However, the rapid emergence of *C auris* globally has led to a hypothesis that this fungus is highly transmissible [4, 11], perhaps due to its ability to survive for extended periods on environmental surfaces [12, 13] or perhaps due to biological features of persistent skin colonization for *C auris* [14, 15], compared to MRSA and CRE for which nares and the gut are thought to be primary colonization sites in humans.

The findings that certain HCP roles are associated with higher rates of *C auris* transfer and that touching the patient leads to more *C auris* transfer are consistent with previous studies [5, 6, 16–18]. For both MRSA and CRE, we found similarly that physical therapists, occupational therapists, and respiratory therapists had higher rates of bacterial transfer to their gloves and gowns compared to other HCP. Thus, HCP in these groups should be educated about the need for meticulous compliance with gloves and gowns and hand hygiene to avoid self-contamination and possible onward transmission of *C auris.* While touching the patient was associated with higher *C auris* transfer rates than touching only the environment, transfer was detected in 13% of encounters in which the HCP had contact with only the environment.

We found that the number of patient body sites colonized was correlated with *C auris* transfer to HCP gloves and/or gown. For example, compared to patients who were colonized at a single body site, those who were colonized at 5 body sites were 15 times more likely to transmit *C auris.* This correlation between burden and transmission has been found for other pathogens in both acute care and nursing home settings [5, 19, 20]. We also found that the highest *C auris* recovery was from the patient finger webs. This is analogous with a recent study that showed improved consistency of *C auris* colonization screening with an anterior nares and hand composite sample [21]. This stresses the importance of patient bioburden reduction for *C auris.* However, controversy exists about the efficacy of CHG for *C auris* decolonization. Some studies have suggested that CHG is effective against *C auris* and that concentration levels are inversely associated with *C auris* burden [22, 23]. Other studies have shown persistent colonization of patients and nursing home residents despite the use of daily CHG bathing [24–27]. Other studies argue that there is variable efficacy of CHG and other biocides against *C auris* [28]. Our study also had mixed findings. We found that higher CHG concentration on the groin was associated with lower *C auris* bioburden on the groin, but this was not the case for the axilla. This controversy suggests that efforts to improve training and quality of CHG bathing for *C auris*–colonized patients is critical, in addition to the need to identify novel and more effective decolonization agents or combination of decolonization agents [9, 29, 30].

*Candidozyma auris* can persist on inanimate surfaces for prolonged periods and it resists routine disinfectants [13, 28]. The frequency of environmental contamination has been described in numerous other studies [12, 24], and the magnitude of environmental contamination has been shown to be associated with patient fungal burden [12]. We observed a high frequency of contamination of items that were frequently touched by HCP (eg, bed rails, supply carts). This could potentially lead to transmission if transfer was to occur and hand hygiene was not subsequently performed, which reinforces the importance of auditing hand hygiene and appropriate use of gowns and gloves with continual feedback to HCP. It also highlights the insufficiency of once-daily cleaning/disinfection for this high-shed pathogen. Of note, the proportion of samples from the floor was particularly high in our study (45%). HCP do not typically touch the floor with their hands, so this environmental site may not frequently lead to patient-to-patient transmission. However, it is possible that shoes could play a role in transfer of *C auris* in the hospital setting and should be a focus of future research. Auditing of hand hygiene, improving compliance with contact precautions, and decolonization compliance should also be a focus for emerging pathogens such as *C auris.*

Our study is the first to analyze risk factors for transfer of *C auris* to HCP gloves and/or gown. Strengths of the study include its multicenter nature and the consistency with protocols used to study transmission of MRSA and CRE. Despite these strengths, there are limitations to our study. First, observations were conducted primarily during weekdays. Second, the entire surface of the HCP gloves and gowns was not cultured; thus, the percentage of gloves and gowns that became contaminated may be underestimated. We used this methodology because it was identical to that of other studies and allowed us to compare the rates of *C auris* transfer of HCP gloves and/or gown to those observed when studying other organisms. Since HCP sampling was random, we did not track how many HCP were sampled more than once. It is therefore possible that practices of a few specific individuals observed on multiple occasions could be distinctive and could influence results for the group. It should also be acknowledged that our study was not designed to assess whether high rates of *C auris* transfer to gloves and/or gown resulted in subsequent patient transmission.

## CONCLUSIONS

*Candidozyma auris* is frequently transferred to HCP gloves and/or gown during routine care of patients with *C auris*. Extensive colonization at multiple patient body sites and shedding onto environmental surfaces contribute to frequent transfer and risk of transmission to others. These findings highlight the need for *C auris* screening and effective strategies to interrupt transfer of *C auris* to HCP during patient care and prevent *C auris* transmission in healthcare facilities.

## Supplementary Material

ofag582_Supplementary_Data

## Supplementary Data

Supplementary materials are available at *Open Forum Infectious Diseases* online. Consisting of data provided by the authors to benefit the reader, the posted materials are not copyedited and are the sole responsibility of the authors, so questions or comments should be addressed to the corresponding author.
