# Supplementary material for: Transfer of Candidozyma auris to Healthcare Personnel Gloves and/or Gowns From Patients and the Environment: A Multicenter Cohort Study of 109 Patients
Source: Open Forum Infect Dis. 2026 Sep 8;13(9):ofag582. doi: 10.1093/ofid/ofag582 (PMC13598334; doi:10.1093/ofid/ofag582)
Supplement: Supplementary file 1 [file ofag582_supplementary_data.docx]

**SUPPLEMENTAL MATERIALS**

**METHODS:**

**STANDARD OPERATING PROCEDURE FOR DATA COLLECTION**

**Title:** Patient and organism factors that increase the spread of antibiotic-resistant material in the hospital

**IRB#:** HP-00066759

**Brief Study Description:** We will perform a multi-site cohort study to determine which patient risk factors, and which healthcare worker-patient interactions lead to greater transmission of *Candida auris*.

**Objective:** The primary objective of this study is to conduct a multicenter cohort study to understand which risk factors, such as patient characteristics, patient care and environment interactions, and which healthcare personnel characteristics are more likely to lead to *Candida auris* transmission from patient to gloves or gowns.

**Overall study:**

Enroll 140 patients with *Candida auris* across multiple acute care hospitals in the United States.

## Aims

Aim 1. Evaluate the association between the type of healthcare personnel, contact with the patient or environment, and the specific activities performed in the room with the contamination of healthcare personnel gloves or gowns with *C. auris*.

Aim 2a. Assess the association between the patient’s bioburden of *C. auris* and glove or gown contamination.

Aim 2b. Assess the association between the concentration of chlorhexidine gluconate (CHG) on the patient’s skin and glove or gown contamination.

Aim 3. Evaluate the association between proportion of environmental surfaces in the patient’s room positive for *C. auris* with the contamination of healthcare personnel gloves or gowns with *C. auris*.

**Description of Patients**

1) All hospital inpatients known to be on contact precautions for *Candida auris*
A. **Procedure for Identifying Cases**
1) Data collection will occur daily, Monday to Friday (Friday enrollment for Maryland only). This may vary by site/staffing.
   a. Check daily for patients currently on isolation for *C. auris*. Developing a relationship with the Infection Preventionist(s) at your hospital is very helpful.
   b. Exclude patients who have already participated in the study.
      i. Once you identify a patient, check the patient’s medical record to confirm that the patient is on isolation for *C. auris* to verify the patient’s location. Record the culture information on the Patient Information Form.
2) Before going to the patient’s room, ensure that you have necessary documents and supplies (see below).
3) Complete the Patient Information Form using the patient chart and/or asking the patient’s nurse, as necessary.
B. **Supplies needed per patient:**
1) BBL flocked swabs (x20)
2) E-swabs (x5)
3) Arrowhead swabs (x2) + container (x2) + template (x2)
4) Sponge sticks (x8)
5) Stencils (x8)
6) Biohazard bags
7) Swab labels
8) Verbal script for HCW
9) Patient Information Form
10) 10 copies of HCW Observation Form
11) Patient Culture Data Collection Form

C. **Procedure for swabbing healthcare worker gowns and gloves** (see training video)

**Supplies:** BBL flocked swabs (x20), swab labels, 10 copies of HCW Observation Form, verbal HCW script, biohazard bag

1) Use verbal script to consent healthcare worker prior to room entry for willingness to have their gown and gloves swabbed upon exiting the room; remind HCW to proceed directly for swabbing before removing gown and glove or performing hand hygiene. **Also remind HCW that if they are double gloved and plan to remove outer gloves at any time to alert study staff so swabbing can be completed prior to removal of outer gloves and gown.**
2) Prepare two swabs by removing the swabs from the outside packaging and placing the swab into the tube, being careful to only touch the plastic cap of the swab. Label one swab for gloves and one swab for gown. Write today’s date on the swab label.

7/7/2022

**HCWID**

3) If the patient is awake and alert, inform the patient about the study and explain swabs to be collected. Reassure the patient that the study will not in any way affect the care they receive.
4) Complete the corresponding information (HCW ID, observation date, entry time and provider type) on HCW Samples Data Collection Sheet.
5) Observe the HCW’s activities in the room using HCW Samples Data Collection Sheet. Place a checkmark next to each item touched by the HCW or activity performed by the HCW.
6) When the HCW is finished and ready to remove his/her gloves or gown:
   a. Remove the single tipped sterile swab labeled “gloves” from the tube. Sample both gloved hands by swabbing the dorsum of each finger (up and down) 3 times followed by 2 circles of the palms using a twirling motion of the swab (sampling the same way for each healthcare worker is important). Carefully return the swab to the tube and close tightly.
   b. Remove the single tipped sterile swab labeled “gown” from the tube. Starting at the wrist where the gown ends, swab each inner forearm twice then swab a “W” along the beltline of the gown. Carefully return the swab to the culture tube and close tightly.

**NOTE**: If the HCW changes gloves during an observation, the observation is over and both gown and glove should be sampled. Swab the gown at the same time and end the observation. If you miss the glove change, do not continue the observation. Discard this observation form and move on to the next HCW.

7) Place the swabs in the biohazard bag.
8) Record the exit/end time on HCW Observation Form.
9) Swabs must be refrigerated after collection and kept cold during shipping
   a. Store the swabs at 4°C until shipping occurs.
   b. Swabs should arrive in Baltimore within 1 day of collection (i.e next day shipping).
   c. See Shipping Guide for detailed shipping instructions.

______________________________________________________________________________________________________________________

D. **Procedure for collecting environmental samples from patient room** (see surface sampling video).

**Supplies:** Sponge sticks (x8), Stencils (x8), Biohazard bag

To identify the presence or absence of *C. auris* in the environment (Yes/No), you will sample 8 standardized high-touch surfaces in the patient’s room. The high touch surfaces to be sampled are the bedrail, bedside table, sink (default to bathroom sink if patient is mobile), floor (side of bed nearest door), supply cart (if no cart, countertop where supplies stored), vital sign monitor (if no monitor, swab computer keyboard), IV pump, remote/call button using 3M™ Sponge-Sticks. Do not use substitutions other than those listed.

1) If the patient is awake and alert, inform the patient about the study and explain environmental swabs to be collected. Reassure the patient that the study will not in any way affect the care they receive.
2) Prepare the sample collection bags by placing labels on upper right corner of each collection bag.

For each environmental site:

a. Wearing clean gloves, open the disposable template package and the sponge package.
b. Place the disposable template over the area to be sampled.
c. Remove the sterile sponge from its package by pushing the swab upwards and moving the top of the stick beyond the package opening. This will avoid contaminating the bag.
d. Wipe the surface to be sampled using the moistening sterile sponge by laying the widest part of the sponge on the surface, leaving the leading edge slightly lifted. Apply gentle but firm pressure and use and overlapping ‘S’ pattern to cover the entire surface with horizontal strokes.

e. Turn the sponge over and wipe the same area again using vertical ‘S’ strokes.

f. Use the edges of the sponge (narrow sides) to wipe the same area using diagonal ‘S’ strokes.
g. Use the tip of the sponge to wipe the perimeter of the sampling area.

h. Place the sponge back into the sterile bag up to the thumb-stop line on the blue stick. Bend blue stick to break and allow sponge to drop down further into bag. Discard stick and fold bag to close. Fold ends of blue wires inward to securely seal bag.
i. Dispose of the template.
j. Repeat for remaining environmental sites.
k. Store the sponges at 4°C until shipment occurs. See shipping guide for detailed shipping instructions.

E. **Procedure for obtaining patient swabs**

To quantify *C. auris* burden on the patient (CFU/mL), we will use ESwabs™ (Copan Diagnostics, Murrieta, CA) to swab five patient sites: the patient’s bilateral axilla, groin/inguinal fold, finger webs, nares and stool/perirectal. Stool is preferred.

**Supplies:** Eswabs (x5), swab labels, biohazard bag

1) If the patient is awake and alert, inform the patient about the study and explain the patient swabs to be collected. Reassure the patient that the study will not in any way affect the care they receive.
2) Write specimen information on the label and apply patient identification label to tube.
3) Open the swab package by grasping the plastic at the opposite end from the soft tip.
4) Carefully remove the tube from its packaging, leaving the swab tip enclosed in the package to prevent contamination.
5) Pull the swab from its package, being careful not to touch the soft tip. Firmly rub the soft end of the collection swab cross the indicated site at least 3-5 times. Specific instructions for all sites to be swabbed:
   a. **Bilateral axilla:** Swipe back and forth both sides of the swab tip over the left axilla skin surface and then the right (3-5 times per crease), targeting the crease in the skin where the arm meets the body
   b. **Bilateral groin:** Swipe back and forth both sides of the swab tip along the groin/inguinal fold 3-5 times per crease.
   c. **Bilateral finger webs:** Swipe back and forth 3-5 times per finger web.
   d. **Bilateral nares:** Rotate 5 times per nostril.
   e. **Stool (preferred)/Perirectal:** Collect small sample of stool on tip of swab if patient has ostomy, is incontinent, or if stool is visible. If stool collection is not possible, default to perirectal swab. Perirectal swab: Swab around the perirectal area 3-5 times using both sides of the swab tip.
6) For each of the 5 swabs collected: Remove the cap from the swab collection tube, then place the soft end of the collection swab into the tube. Be careful to keep the cap from touching any materials that may contaminate your sample.
7) Snap off the end of the swab at the marked line by bending the plastic handle against the edge of the transport media container.
8) Screw on the tube cap. You may need to adjust it until the snapped end of the swab slides into place in the center of the cap.
9) Store the swabs at 4°C until shipment occurs.
F. **Procedure for obtaining CHG concentration sampling**

We will collect a sample from the skin adjacent to the swabbed axilla and groin areas to quantify CHG levels. A colorimetric, semiquantitative method will be used to estimate CHG concentrations. The CHG sites will be collected **AFTER** the patient swabs.

**Supplies:** 2 Arrowhead bioswabs, 2 carrying containers (blue top), 2 paper stencils

1) Each patient to be sampled from 2 adjacent skin sites **AFTER** you have done the other quantitative swabs:

a)    Upper arm close to axilla sample site (CHG-1)

b)    Thigh area close to Groin sample site (CHG-2)

2) Place one label on each container and make sure that the correct swabs are used for the two skin sites
3) Remove the swab from the packaging, snap the blue scored end to release the water - you will notice the water flowing to the tip of the swab
4) Place the paper stencil with circular test area on the patient's skin as close as possible to the area sampled to obtain the axilla skin sample. Be careful not to sample the same skin area that was used to obtain the axilla sample.
5) Sample the entire surface of skin inside the circle by rubbing the swab back and forth across the skin for 10 seconds.
6) Immediately place the swab **(downward facing)** inside the corresponding pre-labeled container after sample collected.
7) Repeat this procedure on the patient’s skin as close as possible to the area sampled to obtain the groin sample.
8) Reminder to record the time the CHG samples collected on the Patient Quantitative form as well as work with patient’s nurse and/or check electronic medical records to complete the nasal decolonization if completed, date/time of last CHG bath and type of bath sections.
G. **Procedure for shipping study samples**

Please follow shipping instructions found

**Data Collection Forms**

**CDC CANDIDA AURIS STUDY**

**PATIENT INFORMATION FORM**

| REPID (MD only): |  | Study Number: |  | Enrollment Date: |  |
| --- | --- | --- | --- | --- | --- |

**Location information**

| ☐ UM Midtown | ☐ UM-SJMC | ☐ Chicago | Unit/Ward: _________________________ |
| --- | --- | --- | --- |
| ☐ UM Capital Regional | ☐ UM-BWMC | ☐ UCI | Room: ______________________________ |
| ☐ UMMC | ☐ UM-Charles Reg | ☐ New York | ICU: ☐ Yes ☐ No |

☐ UM-Chestertown

**Culture information**

| *C. auris* culture date: ________________________________  Final result date: ____________________________________ | Culture source | |
| --- | --- | --- |
|  | ☐ Axilla surveillance swab  ☐ Inguinal/groin surveillance swab  ☐ Finger web surveillance swab  ☐ Wound | ☐ Blood  ☐ Sputum  ☐ Urine  ☐ Nares  ☐ Other (specify): _______________________ |

| **Date** | **Artificial airway (trach/ET tube)** | **Wound** | **Foley** | **Intravascular Catheter (central or PICC line)** | **Chest Tube** | **Surgical Drain** | **Diarrhea** | **Rectal Tube** | **NG Tube** |
| --- | --- | --- | --- | --- | --- | --- | --- | --- | --- |
|  | ☐ | ☐ | ☐ | ☐ | ☐ | ☐ | ☐ | ☐ | ☐ |
|  | ☐ | ☐ | ☐ | ☐ | ☐ | ☐ | ☐ | ☐ | ☐ |
|  | ☐ | ☐ | ☐ | ☐ | ☐ | ☐ | ☐ | ☐ | ☐ |
|  | ☐ | ☐ | ☐ | ☐ | ☐ | ☐ | ☐ | ☐ | ☐ |
|  | ☐ | ☐ | ☐ | ☐ | ☐ | ☐ | ☐ | ☐ | ☐ |
|  | ☐ | ☐ | ☐ | ☐ | ☐ | ☐ | ☐ | ☐ | ☐ |

| **HCW cultures obtained** | |  | **Environmental cultures obtained** | |
| --- | --- | --- | --- | --- |
| **HCW #** | **Date** |  | **ENV #** | **Date** |
| H01 |  |  | EV1 – BEDRAIL |  |
| H02 |  |  | EV2 – BEDSIDE TABLE |  |
| H03 |  |  | EV3 – SINK (default to bathroom sink if patient is mobile) |  |
| H04 |  |  | EV4 – FLOOR (side of bed nearest door) |  |
| H05 |  |  | EV5 – SUPPLY CART (if no cart, countertop where supplies stored) |  |
| H06 |  |  | EV6 – VITALS MONITOR (if no monitor, swab computer keyboard) |  |
| H07 |  |  | EV7 – IV PUMP |  |
| H08 |  |  | EV8 – REMOTE/CALL BUTTON |  |
| H09 |  |  |  | |
| H10 |  |  |  |  |

**CDC CANDIDA AURIS STUDY**

**Observation Form**

**HCW SAMPLES DATA COLLECTION SHEET**

| **HCWID:** |  | | | | |  |  |  |
| --- | --- | --- | --- | --- | --- | --- | --- | --- |
| **OBSERVATION DATE:** | | |  |  |  | |  |  |
| **ENTRY TIME:** | |  | XXXXXX |  | **EXIT/END TIME:** | |  |  |

**NOTE**: If the HCW changes gloves during an observation, swab the first pair of gloves before removal. Swab the gown at the same time and end the observation. If you miss the glove change, *do not continue the observation*. Discard this observation form and move on to the next HCW. Activities observed on this form should reflect what the HCW did while wearing the same pair of gloves and gown.

| **PROVIDER TYPE:** | | | | | |
| --- | --- | --- | --- | --- | --- |
| ☐ | Nurse | ☐ | Respiratory Tech | ☐ | MD/Nurse practitioner |
| ☐ | Occupational/Physical Therapy | ☐ | Patient Care Tech (PCT) | ☐ | Environmental Services |
| ☐ | Other (specify): | | | |  |

| **ENVIRONMENTAL CONTACT** | |  | **PATIENT CONTACT** | |
| --- | --- | --- | --- | --- |
| ☐ | Sink |  | ☐ | Physical exam |
| ☐ | Bed Rail |  | ☐ | Wound dressing |
| ☐ | Bedding |  | ☐ | Bathing/hygiene |
| ☐ | Bedside Table |  | ☐ | Catheter/drain |
| ☐ | Vital Sign Monitor |  | ☐ | ETT/trach |
| ☐ | Supply Cart |  | ☐ | Vital signs |
| ☐ | Lift |  | ☐ | Giving meds (oral) |
| ☐ | IV Pump |  | ☐ | IV tubing/IV meds |
| ☐ | Ventilator |  | ☐ | Transfer in/out of bed |
| ☐ | Curtain |  | ☐ | Blood draw |
| ☐ | Trash |  | ☐ | Glucose monitoring |
| ☐ | Computer |  | ☐ | Rectal tube/bag |
| ☐ | Barcode scanner |  | ☐ | Suctioning |
| ☐ | Call button/remote |  |  |  |
| ☐ | Bathroom surfaces |  |  |  |
| ☐ | Other (specify below) |  | ☐ | Other (specify below) |
|  | |  |  | |

**CDC CANDIDA AURIS STUDY**

**Patient Quantitative Culture Data Collection Form**

STUDY ID: _______________________________

Date of last nasal decolonization: _______________________________

Product used for nasal decolonization:	Mupirocin	Povidone-iodine	Other_______________

Date and time of last CHG bath: _______________________________

Type of bath:	liquid soap	cloth wipe	foam

If patient REFUSED ALL, check here:

|  | **Date obtained** | **Time obtained (CHG swabs only)** | **Not obtained** |
| --- | --- | --- | --- |
| **Axilla** (swipe back and forth 3-5 times per armpit) |  |  |  |
| **Groin/Inguinal fold** (swipe back and forth 3-5 times per crease) |  |  |  |
| **Finger webs** (swipe back and forth 3-5 times per finger web) |  |  |  |
| **Nares** (rotate 5 times per nostril) |  |  |  |
| **Stool or Perirectal:**  Stool if patient has Ostomy (collect small sample of stool on tip of swab) – If no Ostomy, default to Perirectal swab |  |  |  |
| **Axilla** **– CHG** (place stencil adjacent to axilla area and rub swab back and forth for 10 seconds) |  |  |  |
| **Groin – CHG** (place stencil adjacent to groin area and rub swab back and forth for 10 seconds) |  |  |  |

**Supplies Needed:	E-swabs X 5

Arrowhead swabs X 2

Arrowhead swab container X2

**RESULTS**

**Table 1**. Patient samples culture-positive for *C. auris* at the time of enrollment (N=109 patients)

|  | **Axilla** | **Finger** | **Groin** | **Nares** | **Perirectal** | **Stool** | **Number of patients**  **n (%)**  **(N=109)** | **Transfer of *C. auris***  **n/N (%)**  **(N=1083)** |
| --- | --- | --- | --- | --- | --- | --- | --- | --- |
| **One body site** |  | | | | | | **17 (15.5)** | **9/168 (5.4)** |
|  |  |  |  |  |  |  | 3 | 0/30 (0) |
|  |  |  |  |  |  |  | 4 | 5/40 (12.5) |
|  |  |  |  |  |  |  | 4 | 1/40 (2.5) |
|  |  |  |  |  |  |  | 2 | 0/20 (0) |
|  |  |  |  |  |  |  | 3 | 3/30 (10.0) |
|  |  |  |  |  |  |  | 1 | 0/10 (0) |
| **Two body sites** |  | | | | | | **14 (12.8)** | **12/139 (8.6)** |
|  |  |  |  |  |  |  | 2 | 3/20 (15.0) |
|  |  |  |  |  |  |  | 3 | 2/29 (6.9) |
|  |  |  |  |  |  |  | 0 | 0 (0) |
|  |  |  |  |  |  |  | 2 | 1/20 (5.0) |
|  |  |  |  |  |  |  | 0 | 0 (0) |
|  |  |  |  |  |  |  | 1 | 1/10 (10.0) |
|  |  |  |  |  |  |  | 1 | 0/10 (0) |
|  |  |  |  |  |  |  | 1 | 0/10 (0) |
|  |  |  |  |  |  |  | 0 | 0 (0) |
|  |  |  |  |  |  |  | 1 | 1/10 (10.0) |
|  |  |  |  |  |  |  | 1 | 4/10 (40.0) |
|  |  |  |  |  |  |  | 1 | 0/10 (0) |
|  |  |  |  |  |  |  | 1 | 0/10 (0) |
|  |  |  |  |  |  |  | 0 | 0 (0) |
|  |  |  |  |  |  |  | 0 | 0 (0) |
| **Three body sites** |  | | | | | | **14 (12.8)** | **22/137 (16.1)** |
|  |  |  |  |  |  |  | 1 | 0/10 (0) |
|  |  |  |  |  |  |  | 0 | 0 (0) |
|  |  |  |  |  |  |  | 0 | 0 (0) |
|  |  |  |  |  |  |  | 1 | 0/10 (0) |
|  |  |  |  |  |  |  | 0 | 0 (0) |
|  |  |  |  |  |  |  | 0 | 0 (0) |
|  |  |  |  |  |  |  | 0 | 0 (0) |
|  |  |  |  |  |  |  | 0 | 0/10 (0) |
|  |  |  |  |  |  |  | 1 | 0/10 (0) |
|  |  |  |  |  |  |  | 1 | 0/10 (0) |
|  |  |  |  |  |  |  | 1 | 2/9 (22.0) |
|  |  |  |  |  |  |  | 2 | 8/20 (40.0) |
|  |  |  |  |  |  |  | 0 | 0 (0) |
|  |  |  |  |  |  |  | 2 | 4/18 (22.2) |
|  |  |  |  |  |  |  | 2 | 6/20 (30.0) |
|  |  |  |  |  |  |  | 0 | 0 (0) |
|  |  |  |  |  |  |  | 3 | 2/30 (6.6) |
|  |  |  |  |  |  |  | 0 | 0 (0) |
|  |  |  |  |  |  |  | 0 | 0 (0) |
|  |  |  |  |  |  |  | 0 | 0 (0) |
| **Four body sites** |  | | | | | | **28 (25.7)** | **107/279 (38.4)** |
|  |  |  |  |  |  |  | 7 | 25/70 (35.7) |
|  |  |  |  |  |  |  | 7 | 27/69 (39.1) |
|  |  |  |  |  |  |  | 3 | 14/30 (46.6) |
|  |  |  |  |  |  |  | 0 | 0 (0) |
|  |  |  |  |  |  |  | 0 | 0 (0) |
|  |  |  |  |  |  |  | 2 | 8/20 (40.0) |
|  |  |  |  |  |  |  | 0 | 0 (0) |
|  |  |  |  |  |  |  | 7 | 27/69 (39.1) |
|  |  |  |  |  |  |  | 2 | 6/20 (30.0) |
|  |  |  |  |  |  |  | 0 | 0 (0) |
|  |  |  |  |  |  |  | 0 | 0 (0) |
|  |  |  |  |  |  |  | 0 | 0 (0) |
|  |  |  |  |  |  |  | 0 | 0 (0) |
|  |  |  |  |  |  |  | 0 | 0 (0) |
|  |  |  |  |  |  |  | 0 | 0 (0) |
| **Five body sites** |  | | | | | | **36 (33.0)** | **166/360 (46.1)** |
|  |  |  |  |  |  |  | 26 | 122/260 (46.9) |
|  |  |  |  |  |  |  | 10 | 43/100 (43.0) |
|  |  |  |  |  |  |  | 0 | 0 (0) |
|  |  |  |  |  |  |  | 0 | 0 (0) |
|  |  |  |  |  |  |  | 0 | 0 (0) |
|  |  |  |  |  |  |  | 0 | 0 (0) |
| **Six body sites** |  | | | | | | 0 | 0 (0) |
|  |  |  |  |  |  |  | 0 | 0 (0) |
